# From Idiopathic Pericardial Hemorrhage to Mycotic Aortic Aneurysm: A Case Report

**DOI:** 10.7759/cureus.100751

**Published:** 2026-01-04

**Authors:** Jorge Licano, Basel M Taha, Sameen F Jawadi, Umair A Khan, Carolina D Ponce-Orellana

**Affiliations:** 1 Department of Internal Medicine, University of New Mexico School of Medicine, Albuquerque, USA; 2 School of Medicine, University of New Mexico School of Medicine, Albuquerque, USA

**Keywords:** ascending aortic aneurysm, endovascular infection, hemorrhagic cardiac tamponade, hemorrhagic pericarditis, mycotic aortic aneurysm, staphylococcus capitis

## Abstract

Mycotic aneurysms are a dilatation of an artery due to infection of the arterial wall. These can develop in various locations. Common pathogens involved are *Streptococcus* species and *Staphylococcus aureus*. Yet, there are no documented cases attributed to *Staphylococcus capitis*. A 47-year-old woman presented with intermittent chest pain. A diagnosis of acute pericarditis with moderate pericardial effusion was made based on transthoracic echocardiogram (TTE) findings. Evaluations for infection, vasculitis, and autoimmune conditions were negative, at which time the etiology of pericarditis was deemed idiopathic. Computed tomography (CT) showed no aortic findings. She was subsequently started on aspirin and colchicine therapy. Patients symptoms then resolved, and an improved small pericardial effusion was seen on repeat TTE. Three days after discharge, she returned in cardiogenic shock due to a large tamponade. New CT imaging demonstrated a 1.5 cm ascending aorta aneurysm. Pericardiocentesis was deferred due to concern for rapid hemopericardium reaccumulation. Aortogram confirmed CT findings of ascending aorta outpouching above the bifurcation of the right coronary artery. Emergent replacement of the ascending aorta with drainage of pericardial effusion was then performed with intraoperative inspection verifying imaging findings. Blood cultures were negative. However, the surgical specimen grew *S. capitis*, a rare and slow-growing organism with scarce literature as it pertains to mycotic aortic aneurysms. The patient ultimately recovered from surgery and was discharged on appropriate antibiotics.

## Introduction

Mycotic aortic aneurysms (MAAs) develop via microbial inoculation of aortic endothelium during bacteremia. Upon pathogen invasion, the intimal and medial layers of the vessel wall can degrade, leading to aneurysm formation [1]. Although uncommon in occurrence, they carry a poor prognosis due to their rapid growth and risk of rupture, with some studies revealing 38-42% mortality in patients presenting with ruptured aneurysms [2]. The most commonly involved pathogens include *Staphylococcus aureus* and *Staphylococcus epidermidis*, among many others [3]. 

Prompt intervention with surgical or endovascular approaches is essential to prevent complications [4]. However, diagnosis for this rare entity can be challenging, with positive blood culture as a prerequisite being a point of disagreement [5]. The American Heart Association (AHA) currently describes four criteria to aid in diagnosis, including evidence of systemic infection, positive blood cultures, imaging findings, and contiguous spread from an adjacent infection [6]. Yet, to our knowledge, there is no established algorithm, and no single criterion is sufficient for diagnosis. We discuss the case of a patient with pericardial effusion caused by a MAA due to *Staphylococcus capitis*.

This case was previously presented as a meeting abstract at the 2025 American College of Cardiology Scientific Sessions Meeting on March 29, 2025. 

## Case presentation

A 47-year-old female with prior tobacco use, currently in remission, presented with diaphoresis, near syncope, and acute chest pain that improved when leaning forward. She denied any previous cardiac history, including coronary artery disease, heart failure, or hypertension. Computed tomography (CT) with contrast performed in the emergency room demonstrated a small pericardial effusion with no aortic or pulmonary findings. A clinical diagnosis of acute pericarditis without signs of cardiogenic shock was made.

Transthoracic echocardiogram (TTE) confirmed a moderate pericardial effusion with no tamponade physiology. A pericardiocentesis was recommended due to her near syncope presentation. The patient declined this procedure after risk and benefits were discussed in the setting of a borderline hemodynamic profile. Therapy for pericarditis with high-dose aspirin and colchicine was then initiated. A full infectious and autoimmune workup was completed, which was inconclusive. This included blood cultures, hepatitis screening, viral essays, anti-nuclear antibody, C-reactive protein (CRP), and erythrocyte sedimentation rate (ESR), which were all negative. Her symptoms subsequently improved, and exercise tolerance returned to baseline without pre-syncope upon physical therapy evaluation. She was then discharged on a three-month pericarditis treatment plan with close follow-up and strict return precautions. 

**Figure 1:**
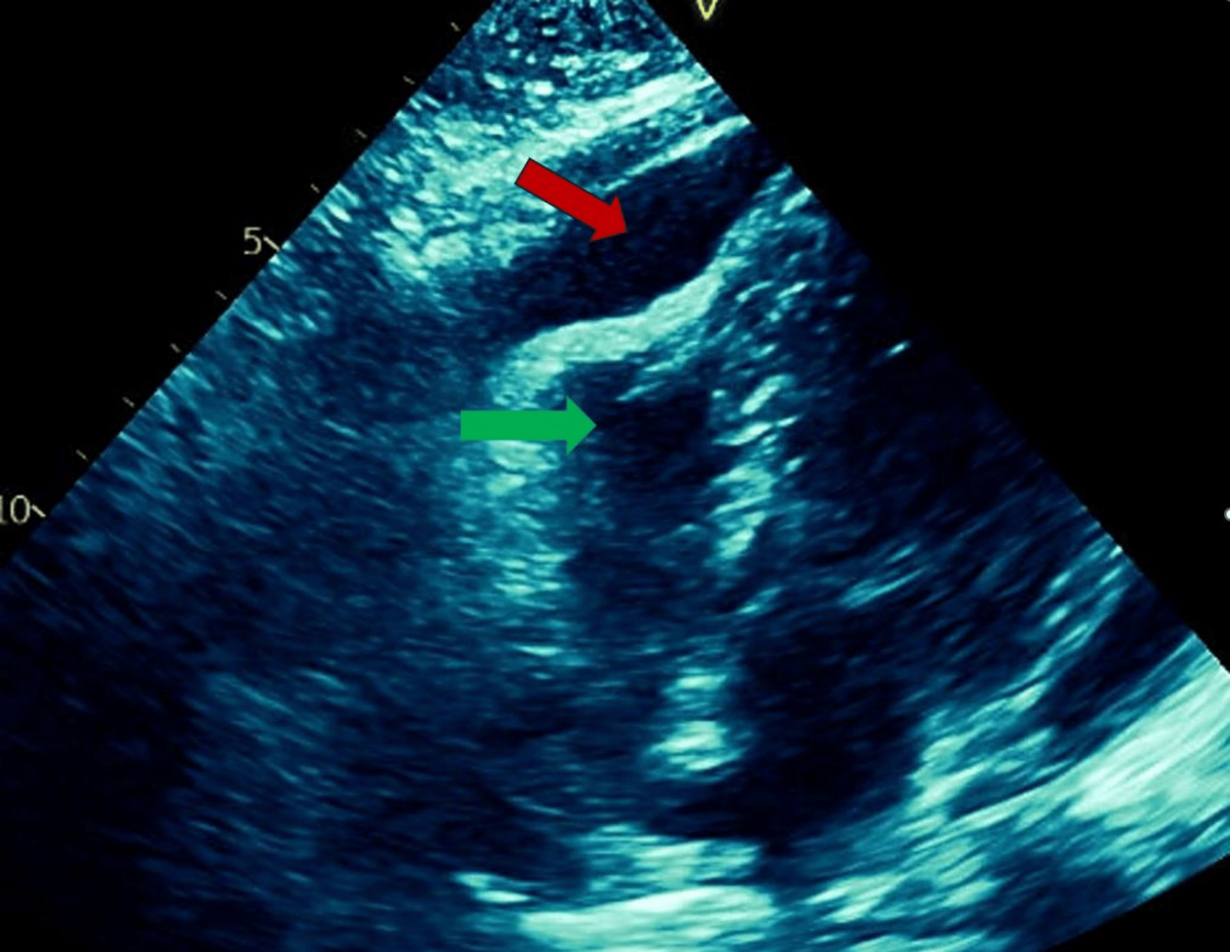
Transthoracic echocardiogram with red arrow demonstrating pericardial effusion causing the right ventricle to collapse (green arrow).

Conversely, the patient returned to the hospital three days after her discharge. She presented with shortness of breath, a syncopal episode, diaphoresis, and worsened chest pain. Vital signs were notable for hypotension, tachycardia, and tachypnea. All of her laboratory results were normal, other than a decreased hemoglobin of 11.3 g/dL and hypokalemia of 3.1 mEq/L. Physical exam revealed muffled heart sounds without apparent jugular venous distention. She was consequently admitted to the intensive care unit for cardiac tamponade requiring intravenous fluid resuscitation and pressor support. A bedside TTE demonstrated a moderate-to-large pericardial effusion with right ventricle diastolic collapse (Figure 1).

**Figure 2:**
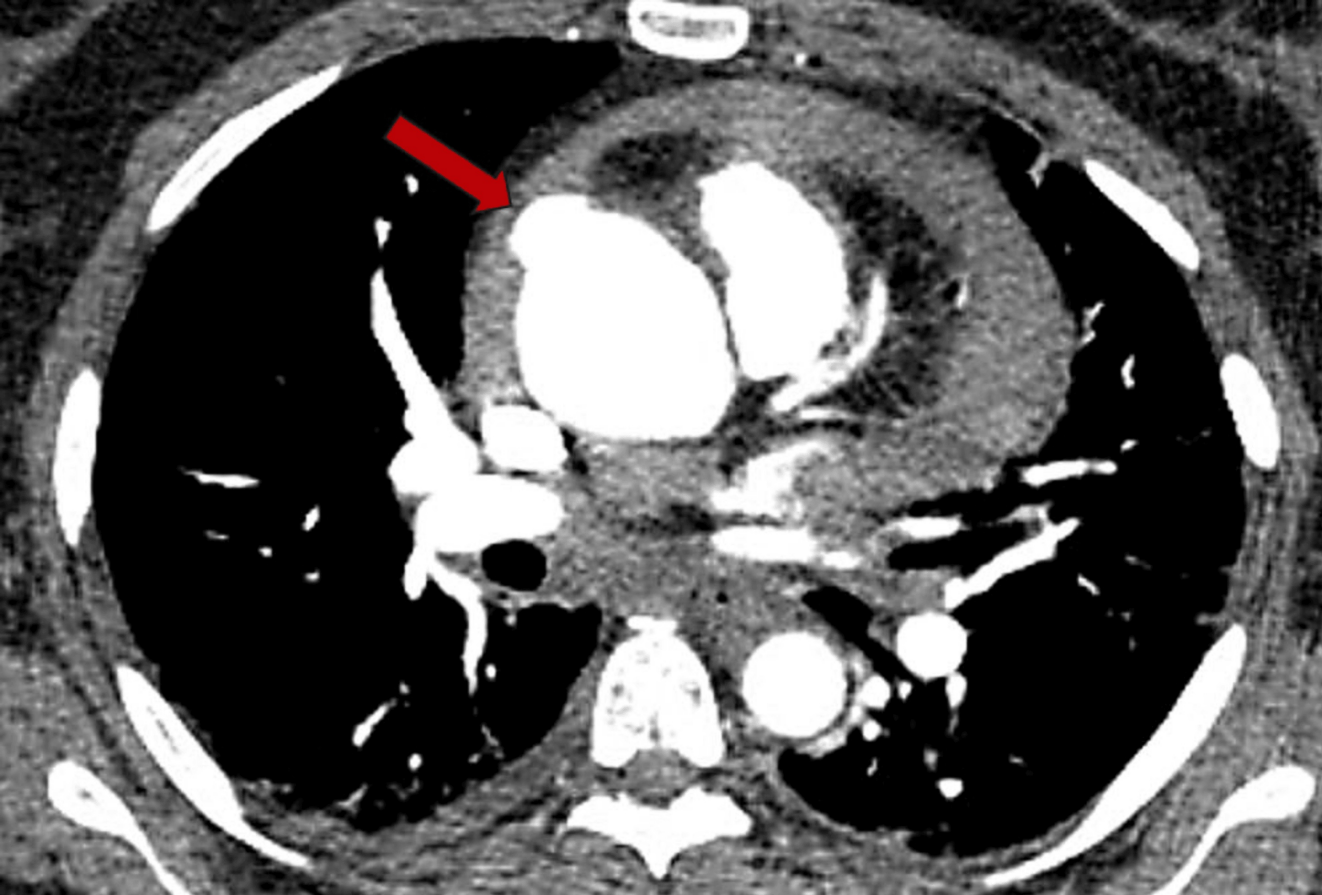
Axial CT chest angiogram revealing small ascending aortic aneurysm (red arrow).

A resident generated a preliminary report for a repeat CT angiography of the chest, which was initially negative. However, additional review with the attending radiologist revealed evidence concerning for a 1.5 cm ascending aorta aneurysm superior to the right coronary artery (Figure 2). Aspirin and colchicine were discontinued at this time pending possible cardiothoracic surgery intervention.

**Figure 3:**
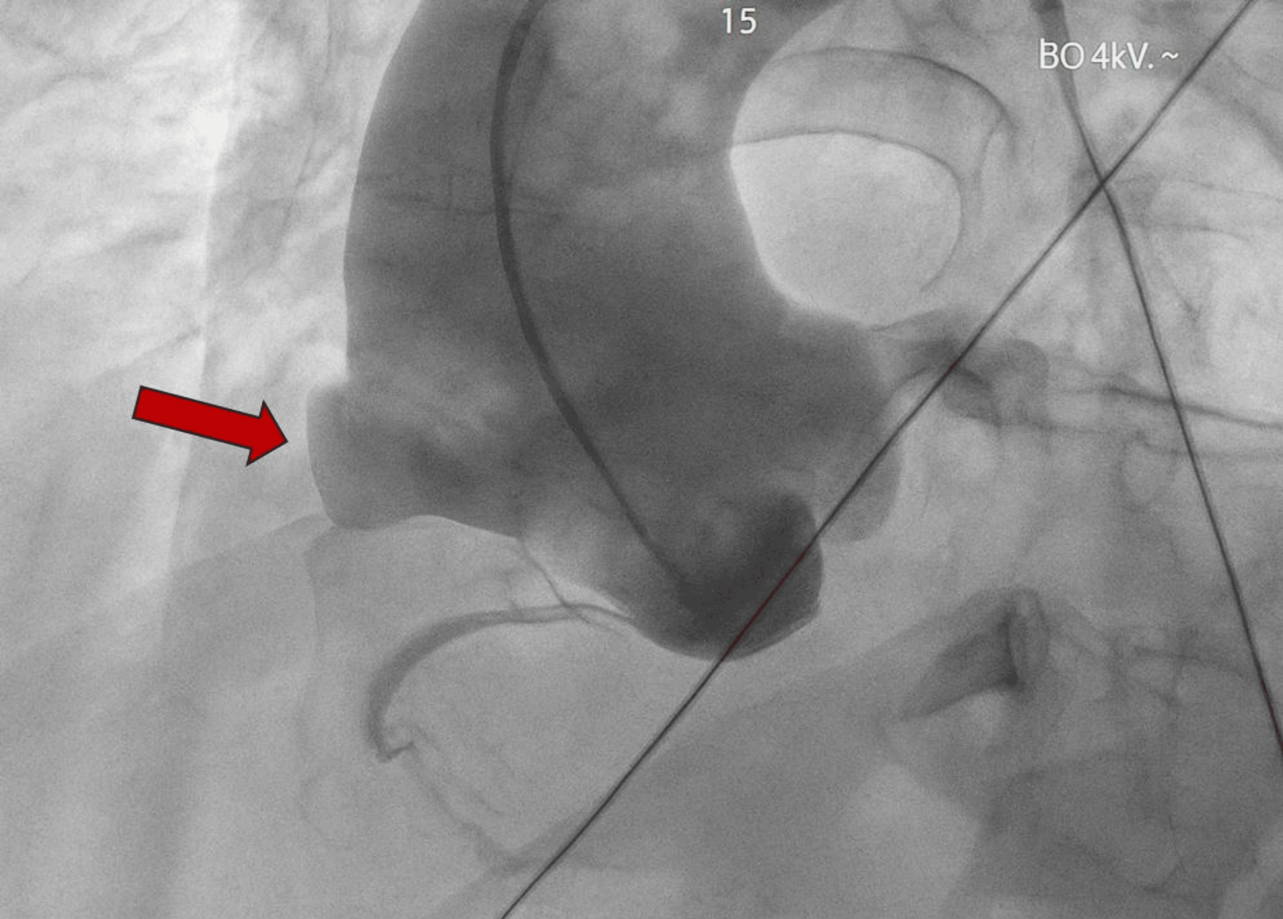
Anteroposterior projection of aortogram showing ascending aortic aneurysm (red arrow) proximal to the right coronary artery bifurcation.

The patient was then taken to the cardiac catheterization lab for coronary angiography and aortogram to better characterize the ascending aneurysm. This demonstrated patent coronary arteries and a dilated ascending aorta with a right-sided “outpouching” above the bifurcation of the right coronary artery (Figure 3). It is important to note that no extravasation was appreciated. Pericardiocentesis was deferred due to concern that blood in the pericardium would quickly reaccumulate from the connecting aneurysm.

**Figure 4:**
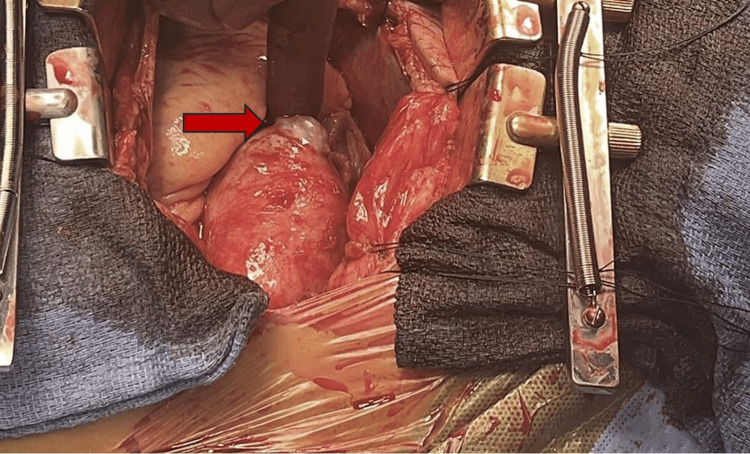
Intraoperative visualization of 1.5 cm ascending aortic aneurysm

Cardiothoracic surgery then performed an emergency sternotomy for ascending aortic aneurysm repair. The pericardium was found to be under tension, and hemorrhagic pericardial effusion drainage resulted in a 30 mmHg improvement in systolic blood pressure. Intraoperative findings were also significant for a 1-2 cm penetrating ulcer of the ascending aorta, about 2 cm distal to the right coronary artery origin (Figure 4). There was a microperforation within this outpouching with active bleeding into the pericardial space.

Other findings included a dilated ascending aorta and minimal nodularity of the aortic valve. Conversely, the adjacent aorta appeared atherosclerotic and discolored without dissection or signs of aortitis. Thus, simply patching the affected area did not seem adequate. The ascending aorta was therefore resected from the sinotubular junction, and surgical pathology was sent for evaluation and culture.

A 24 mm graft was then beveled appropriately, sewn to the sinotubular junction using running 4.0 Prolene suture, and anastomosed distally to the aorta just proximal to the takeoff of the innominate artery. The graft size was excellent for the aorta, and significant tailoring or additional suture placement was not necessary. The patient returned to the intensive care unit following surgery. She remained intubated and paralyzed overnight with successful extubation on postoperative day one. There was no vasopressor requirement in the postoperative period. Surgical pathology was negative for aortitis, although surgical culture grew *S. capitus*. Infectious Diseases was then consulted, which recommended starting the patient on cefadroxil based on culture sensitivities. The patient was subsequently discharged on postoperative day six with multidisciplinary outpatient follow-up.

## Discussion

Mycotic aortic aneurysm (MAA) is a rare condition that is associated with high mortality. It's a term that was initially coined by Osler to describe aneurysms associated with infectious endocarditis, typically bacterial in etiology [7]. There have been significant debates on the terminology and lack of universal agreement on how to define this rare pathology [1,7]. It is most commonly defined as an infectious breach in the wall of an artery, leading to a saccular outpouching that is contiguous with the arterial lumen [6]. It is characterized as a pseudoaneurysm, given that it does not involve all layers of the aorta [7]. Furthermore, four mechanisms have been proposed to cause infectious aneurysms: (i) Septic emboli that occlude the blood vessel, leading to bacterial extension into the vessel wall, resulting in weakening of the wall and aneurysm formation, (ii) Direct extension of the infection to the arterial wall from a contiguous point, (iii) Hematogenous spread during bacteremia leading to bacteria entering an atherosclerotic plaque or infecting a preexisting aneurysm, and (iv) Direct blood vessel trauma or contamination such as penetrating trauma, intravenous drug use, or post procedural complications [8-11].

Moreover, the incidence of MAA has been difficult to determine, and most published literature likely underestimates its true incidence. A study from Mayo Clinic between 1925 and 1954 found only six MAA among 20,000 autopsies [12]. Meanwhile, a different study from Boston City Hospital estimated the incidence to be 1.5% [13]. Due to this low incidence and its nonspecific clinical presentation, diagnoses of an MAA can be challenging. For instance, blood cultures can be negative in 25-50% of MAA cases, and leukocytosis can be nonspecific, occurring in 65-85% of patients [6]. For these reasons, the AHA highlights that the diagnosis of MAA depends on a combination of factors, including an index of suspicion, recognition of clinical presentation, physical exam findings, and objective data such as laboratory tests and imaging [14].

The microbiological variety of MAA-causing organisms is changing [11]. For example, gram-negative bacilli such as nontyphoidal *Salmonella* have been documented to cause MAA [11]. Although *S. aureus* is the most common cause of MAA, coagulase-negative staphylococci, which include *S. capitis*, are increasingly recognized in arterial infections [6,15]. *S. capitis* is also known for causing prosthetic joint and soft tissue infections, especially in immunocompromised individuals [16]. Yet to our knowledge, there are only a few cases documented of this organism causing arterial infections, including infective endocarditis [17]. 

Our case demonstrates the challenges of MAA diagnosis. In this particular instance, the patient initially presented with acute pericarditis, which initially responded to aspirin and colchicine. The occurrence of pericarditis likely represented early hemorrhagic leakage from the aortic aneurysm, resulting in pericardial irritation. The patient then returned with cardiogenic shock secondary to tamponade physiology, with repeat imaging revealing a saccular aneurysm of the ascending aorta. This is a finding that was not evident on the initial CT scan, representing a presumed growth of the aneurysm compared to her initial presentation. The rapid growth between admission underscores the unpredictable nature of MAA, which can expand quickly due to ongoing inflammation and arterial wall damage. This condition is prone to progression irrespective of infection severity, explaining its high risk of mortality, rupture, and tamponade complications.

This case also highlights that traditional risk factors for MAA, including recent aortic surgery, immunosuppression, infective endocarditis, or bacteremia, do not need to be present for this rare condition to occur, further contributing to the diagnostic complexity. Moreover, it emphasizes that a more robust diagnostic criterion is needed to avoid delays in the diagnosis of this condition. The isolation of *S. capitis* from the surgical specimen also adds a unique dimension to this case. The involvement of this organism in MAA is rare, yet it emphasizes the importance of identifying subtle pathogens, especially in cases where blood cultures remain negative. This organism’s clinical behavior may contribute to delayed systemic manifestations, posing additional diagnostic challenges.

Interestingly, the surgical specimen revealed significant atherosclerosis in the ascending aorta, an unusual finding for the patient’s age and medical history. Atherosclerosis likely played a critical role in predisposing the aorta to infection. As mentioned previously, one of the mechanisms of MAA development is bacterial colonization of atherosclerotic plaque, leading to endothelial damage. Although our patient had a known tobacco use disorder, the extent of atherosclerosis in this case raises questions about whether additional genetic, inflammatory, or environmental factors may have contributed to its development. Lastly, this case also demonstrates that hemorrhagic pericarditis without a clear identifiable etiology should prompt earlier repeat imaging. 

## Conclusions

This case highlights the diagnostic challenges of MAA, particularly when risk factors are absent and when the clinical presentation suggests conditions that are more common. The rapid progression of MAA underscores the unpredictable nature of this condition, emphasizing early recognition to avoid catastrophic outcomes. Early recognition can be aided by multiple imaging modalities to corroborate findings, especially in small aortic aneurysms such as this case. The isolation of *S. capitis* further illustrates the need to consider the involvement of rare organisms, especially when blood cultures are negative. The presence of atherosclerosis likewise raises questions about the role of underlying vascular conditions in the development of MAA. Nevertheless, additional research is warranted to help identify risk factors, imaging guidelines, and a more specific diagnostic criterion. 
